# IMMPACT-recommended outcome measures and tools of assessment in burning mouth syndrome RCTs: an international Delphi survey protocol

**DOI:** 10.1186/s13063-020-04640-4

**Published:** 2020-08-12

**Authors:** B. Carey, A. M. Farag, C. Nasri-Heir, G. D. Klasser, A. Ariyawardana, M. Chmieliauskaite, A. Sardella, C. R. Carlson, C. S. Miller, L. Mejia, F. E. O’Neill, R. Albuquerque

**Affiliations:** 1 Oral Medicine Department, Guy’s and St. Thomas Hospital NHS Foundation Trust, King’s College London, London, UK; 2 Department of Oral Diagnostic Sciences, Faculty of Dentistry, King AbdulAziz University, Jeddah, Saudi Arabia; 3 Division of Oral Medicine, Department of Diagnostic Sciences, Tufts School of Dental Medicine, Boston, MA USA; 4 Department of Diagnostic Sciences, Center for Temporomandibular Disorders and Orofacial Pain, Rutgers School of Dental Medicine, The State University of New Jersey, Newark, NJ USA; 5 Department of Diagnostic Sciences, School of Dentistry, Louisiana State University Health Sciences Center, New Orleans, LA USA; 6 College of Medicine and Dentistry, James Cook University, Cairns, Queensland Australia; 7 Metro South Oral Health, Brisbane, Queensland Australia; 8 Department of Oral and Maxillofacial Medicine and Diagnostic Sciences, School of Dental Medicine, Case Western Reserve University, Cleveland, OH USA; 9 Department of Biomedical, Surgical and Dental Sciences, Unit of Oral Medicine, Oral Pathology and Gerodontology, University of Milan, Milan, Italy; 10 Orofacial Pain Clinic, College of Dentistry, University of Kentucky, Lexington, KY USA; 11 Department of Oral Health Practice, College of Dentistry, University of Kentucky, Lexington, KY USA; 12 College of Dental Medicine, Nova Southeastern University, Fort Lauderdale, FL USA; 13 Department of Oral Surgery, School of Dentistry, University of Liverpool, Liverpool, UK; 14 Department of Oral Medicine, Faculty of Dentistry, Oral Craniofacial Sciences, King’s College London, Floor 22, Guy’s Tower, London, SE1 9RT UK

## Abstract

**Background:**

A core outcome set (COS) represents the agreed minimum set of domains and measurement instruments that should be measured and reported in any clinical trial for a given condition. In BMS randomized controlled trials (RCTs), the outcomes identified in the existing literature regarding the efficacy of therapeutic interventions are numerous and diverse. Although the standardized IMMPACT core outcome domains has been developed for measurement of outcomes in chronic pain RCTs, no BMS-specific COS have been adopted and validated. With the evolving landscape of BMS management end points and the development of new therapies, a consensus on a COS for use in future BMS trials is paramount to reduce heterogeneity in outcome reporting. The aim of this study was to reach a consensus for adopting the standardized Initiative on Methods, Measurement, and Pain Assessment in Clinical Trials (IMMPACT) outcome domains, and their tools of assessment, for burning mouth syndrome (BMS) clinical trials and clinical practice.

**Methods:**

A BMS-specific COS will be developed using the method recommended by the Core Outcome Measures in Effective Trials (COMET) initiative (Registration: http://www.comet-initiative.org/studies/details/1357). Selection of questionnaire outcome measures was informed by the IMMPACT consensus and previous systematic review of RCTs in BMS conducted by the consortium. An international group of clinicians and researchers will be invited to participate in several rounds of a Delphi survey. A consensus meeting will be held with the objective of ratifying the outcomes for inclusion in the COS. A finalized COS explanatory document will be drafted, including all outcomes and measurements as determined by the Delphi rounds and consensus meeting.

**Discussion:**

A COS for the management of BMS will improve the quality of future RCTs, reduce outcome reporting heterogeneity, and facilitate more vigorous data synthesis of management interventions for systematic reviews and meta-analysis. This would ensure enhanced quality evidence for clinical management of the condition.

## Introduction

Randomized controlled trials (RCTs) represent the gold standard for generating evidence on the efficacy of clinical interventions and should incorporate clearly defined outcome measures. The outcome measures identified in published RCTs concerning the efficacy of therapeutic interventions in burning mouth syndrome (BMS) are numerous and inconsistent [1]. To date, no BMS-specific outcome measures exist. BMS is a unique syndrome as occasionally not only pain is experienced, but often other dysesthesias including dry mouth and dysgeusia exist, symptoms currently not captured on existing outcome measures. Also, as BMS affects the oral cavity, it requires certain core outcome domains including functioning (for example, avoidance of food types, frequency of dietary-related discomfort) to be taken this into consideration which could potentially be useful measures of improvement separate to pain. The Initiative on Methods, Measurement, and Pain Assessment in Clinical Trials (IMMPACT) is an expert consensus strategy that provides a set of recommended core outcome domains to be considered when conducting RCTs concerning interventions for chronic pain and includes: pain dimension, physical functioning, emotional functioning, participant’s ratings of global improvement, symptoms and adverse events, and participant’s disposition [2, 3]. This set of outcome domains recommended for chronic pain is highly applicable for BMS and can be adopted when conducting BMS RCTs to ensure consistency and homogeneity across RCTs in assessing management outcomes. A recent systematic review explored the reporting of IMMPACT outcome measures in all BMS RCTs, published after 1994, and identified a paucity of studies reporting on these standardized measures. Poor utilization of accepted outcome measures may limit transparency, impact the interpretation of treatment effects and, subsequently, could create inaccurate evidence-based healthcare decisions [4]. In addition, it limits the ability to compare findings between RCTs and synthesize data for meta-analyses. The ideal core outcome set (COS) should cover biological, psychological, and social aspects affected by chronic pain [5].

The Core Outcome Measures in Effective Trials (COMET) (http://www.comet-initiative.org/) initiative, funded by the MRC North West Hub for Trials Methodology (NWHTMR) and launched in 2010, addresses the problem of heterogeneity in RCT outcome measures by developing a guideline for the creation of a COS [6]. A COS represents the agreed minimum set of domains and measurement instruments that should be measured and reported in any clinical trial for a given condition to ensure comparable outcome assessment [7]. With the evolving landscape of BMS management end points and the development of new therapies, a consensus agreement on a COS for use in future BMS trials, based on the existing IMMPACT core outcomes, is of vital importance. The Delphi method, which uses a series of sequential questionnaires sent to participants who are chosen for their expertise in their field, has been used in multiple studies with the aim of developing a COS when no consensus or guidelines exist [8–10].

It is expected that the COS will always be collected and reported, but is not so restrictive in negating clinicians and researchers from exploring additional outcomes. Implementation of a successful COS in BMS RCTs will provide a minimum outcome-reporting standard and should reduce heterogeneity in reporting findings. Reducing heterogeneity in outcome reporting will overcome the limitations in the interpretability of results and enable integration of data in systematic reviews and meta-analyses.

To the best of our knowledge, no studies have been conducted to develop consensus on the minimum COS that should be measured and reported in BMS RCTs. Consequently, this study aims to describe the protocol for developing, and reaching expert consensus, for a COS that can be recommended for inclusion in future clinical trials on BMS management strategies.

## Methods

A systematic literature review has previously been conducted to identify potential efficacy and safety outcomes currently reported in BMS RCTs (i.e., IMMPACT outcome domains) [1]. A search of the COMET initiative database (http://www.comet-initiative.org/) was completed prior to commencing this project. No published or ongoing COS for BMS trials was identified. A Delphi method [11] will be used by an international steering group with the intention of developing a COS, based on the existing IMMPACT outcome domains, for future RCTs concerning the management of BMS. The published recommendations for the development of an international consensus BMS-specific COS using the Delphi technique and checklist will be followed [12, 13]. The study protocol was checked via the COS-STAP (Core Outcome Set-STAndardised Protocol Items) [14] checklist (Additional file 1).

Several consecutive rounds of online questionnaires to generate consensus on the BMS COS will be conducted involving researchers and clinicians, with broad geographical representation, using a three-round Delphi survey, with additional rounds if needed. The anonymity of the Delphi method allows panel members to derive consensus and gives equal weight to all participants, avoids prominent personalities from dictating the consensus, and facilitates international involvement [11]. After each round (iteration), the group responses are provided to participants who can then reconsider their decision in light of other viewpoints. Identification of additional outcomes important to clinicians with a special interest in BMS will take place by using free response options in the survey rounds. The COS will be ratified in a consensus meeting of global experts. This COS has been listed in the non-database list of the COMET initiative (http://www.comet-initiative.org/studies/details/1357).

### Scope of the BMS COS

This COS is intended as the international standard for clinical trials examining the efficacy of management strategies in adult patients (≥ 18 years) with a clinically defined diagnosis of burning mouth syndrome in accordance with recognized diagnostic criteria such as The International Classification of Orofacial Pain (ICOP) or the International Headache Society (IHS), who will undergo an interventional therapeutic trial in an attempt to manage their oral burning symptoms.

### Objectives

1. To develop a list of BMS reported outcomes based on the IMMPACT recommendations, for use in a Delphi survey
2. To develop a consensus on a preliminary BMS-COS and their tools of assessment using the outcomes collated from objective 1, via a 3-round Delphi survey.
3. Using the preliminary COS, develop a final COS, and their tools of assessment, for use in future BMS RCTs and in clinical practice.
4. Disseminate and promote the implementation of the COS to key stakeholders internationally.

### Stakeholder involvement

Outcomes measured, and their tools of assessments, in clinical trials must be meaningful to healthcare professionals, researchers, and patients to ensure its acceptance and its implementation. In this project, we will conduct surveys, with both qualitative and quantitative responses, with clinicians and researchers in the field of BMS with the following aims:

1. Consensus on outcome measures generated through the IMMPACT recommendations as reported in the recently published systematic review [3].
2. Expansion of this list with items considered important but not captured in the systematic review.
3. Reaching consensus regarding what tools of assessment should be utilized in BMS RCTs for those IMMPACT domains where multiple tools of assessment were recommended.

### Inclusion criteria for participants

- Age ≥ 25 years old
- Ability to read and write in English
- Hold a post-graduate qualification (MS, DMD, DDS, PhD, or equivalent)
- Access to online surveys
- Provided ≥ 5 years clinical care and/or research in BMS.

### Sample size for participants

There is currently no published COS for measuring/monitoring facial pain in trials and clinical practice, though a COS for trigeminal neuralgia is currently in development (http://www.comet-initiative.org/studies/details/1123). Other studies have used 9 to 678 (median 110) participants in their protocol to develop a COS [9, 15–18]. Consequently, with a sample size of 40 to 50, we will reach an adequate representative sample. No sample power calculation is required for this study for two reasons: (1) the lack of previous studies in this specific topic and (2) the goal is to see a pattern in answers and reach expert agreement, rather than statistical significance.

### Dissemination of the survey

Questionnaires will be electronically sent to clinicians/researchers who specialize in oral medicine, orofacial pain, and/or clinical psychology who assess BMS patients. This will include members of oral medicine and orofacial pain societies, such as the European Association of Oral Medicine, British and Irish Society of Oral Medicine, American Academy of Oral Medicine, American Academy of Orofacial Pain, The Oral Medicine Academy of Australia, Australian and New Zealand Academy of Orofacial Pain, and International Association for the Study of Pain. List-serves will be utilized. This allows for a global range of clinical experiences and geographical expertise. Purposive sampling also will be undertaken to identify and prioritize individuals who have published in the field of BMS in the last 15 years. All potential participants will be contacted with an invitation letter outlining the aims and details of the study and the rationale and importance of completing the Delphi approach, along with an invitation to take part via a link to Qualtrics® (https://exchange.tufts.edu/owa/redir.aspx?C=n_lkhDqjBVtUSGmKSH9aT2s1RhsMTaCmbAM_rGV6pLk1WiXgVdrXCA..&URL=https%3a%2f%2ftufts.qualtrics.com%2fjfe%2fform%2fSV_3sn85hA3IIYZxqZ). For each round of the process, participants will have 14 days to complete the survey with e-mail reminders sent out four days before closure. Participants will be masked to the other respondents in the study.

### Data handling

We anticipate an 80% response rate to reach our goal of having at least 32–40 participants in each round. Drop-outs of the study will not be counted during the round which they drop-out. However, their responses from previous rounds will be analyzed. Data will be exported to an Excel spreadsheet for qualitative and quantitative data analysis. NVivo software will be used for qualitative free text data analysis and coding of themes. Data will be secured in encrypted and password protected files. Inductive coding will be used to code open-ended responses in each survey round [19]. A code book to analyze free response data will be developed by reading line-by-line responses from 10 participants and explicit definitions and rules for applying the code will be created; additional codes will be added if needed [19, 20]. Each transcript will be independently coded by two members of the research team to allow for inter-coder reliability (ICR) assessment of agreement throughout the coding process. ICR will be calculated using the Kappa statistic, and members of the research team will meet to resolve discrepancies when a code’s Kappa is less than 0.5 and percent agreement is less than 80% [21].

### Ethical approval and funding

Ethical approval has been obtained from Tufts School of Dental Medicine Boston, Massachusetts (IRB ID: STUDY00000329). There is no funding obtained for this project. Informed consent will be obtained at the start of each round by a tick box stating “I consent, begin the study.” Identifying information will not be collected during the consenting process.

### Detailed method for each Delphi round

#### Delphi round one

We anticipate the Delphi process will consist of three rounds of electronic-based questionnaires conducted between May and December 2020. Background information on the rationale of the development of the COS will be provided. Respondents will initially be asked to indicate the stakeholder group to which they belong and complete questions regarding their professional background and clinical or research experience relevant to BMS. The list of outcomes generated from the systematic review will be formatted into questions with a 5-point Likert scale, from “strongly agree” to “strongly disagree,” to identify outcomes of importance to experts [22]. The first round will include close-ended and open-ended questions. Prior to sending the questionnaires, they will be pilot tested by a small group of clinicians not involved in the study to ensure clarify. Patients will be involved in the development and review of the questionnaires used in this study prior to distribution to study participants.

Consensus is pre-defined as at least 80% of the respondents being in agreement (Table 1) [8, 9, 18, 23]. Agreement is defined as a response of “agree” or “strongly agree” for a given item. If consensus is reached in any of the rounds, then those items will be omitted from rating in the subsequent round. Items that reach consensus in round 1 will be brought forward for round 2 only if there are suggested changes so that they can be rated again in the context of new suggestions.

**Table 1:** Definitions of consensus

Consensus classification	Description	Definition
Consensus in	Outcome should be included in the COS	> 80% respondents scoring as 4 or 5 AND < 15% scoring as 1 or 2
Consensus out	Outcome should be excluded from the COS	50% or fewer scoring 4 or 5 in each stakeholder group
No consensus	Uncertainty regarding importance of outcome	Anything else

The initial items to be used for round 1 were based on IMMPACT consensus outcome domains as specified in the recent systematic review conducted by our group to identify reported outcomes in BMS RCTs. Forty-six items will be included in round 1. Through free text entry, respondents will have the option to clarify opinions for and against inclusion of outcomes and to provide additional suggestions on other relevant clinical outcomes not included in the round 1 questionnaire.

Round 1 will allow the steering group to assess the frequency and mode of the scores of all respondents and use this as a measure of agreement. A large spread is associated with a weaker consensus. Data analysis from round 1 and creation of the subsequent questionnaire for the next round will take place 2 weeks after the response due date. Responses from round 1 will be analyzed by descriptive statistics to summarize the number of participants scoring each outcome and the distribution of scores in a feedback report. Any new clinical outcomes identified from free text responses will be added to Delphi round 2. Non-respondents of the round 1 survey will not be invited to participate in round 2.

#### Delphi round two

Round 2 will be a close-ended questionnaire, with one or two open-ended questions if needed. In round 2, each participant will be provided with the number of respondents and distribution of scores for each outcome generated from round 1. Participants will then be asked to rescore each outcome in light of the group responses. Each outcome will be re-scored on a 5-point Likert scale as previously described. Participants may choose to change their score or not. Changes in score from round-to-round will be documented. Responses from round 2 will be analyzed using descriptive statistics. Participants will also be asked to score any new clinical outcomes identified in round 1. Participants will have the opportunity for a free response item if they would like to provide additional opinions.

#### Delphi round three

Similar to round 2, round 3 will be a close-ended questionnaire. A feedback report of items which reached consensus, response rate, and frequency and mode of responses will be provided to participants. Each outcome that failed to reach consensus in the previous rounds will be presented and re-scored on a 5-point Likert scale. If consensus is reached, the new COS for BMS RCTs will then be presented as a group for voting. If consensus is reached on additional outcomes, they will be added and the final COS proposed will be presented for final voting. Outcomes not meeting the definitions of inclusion and exclusion, as discussed above, will be classified as lack of consensus. Study participants will also be asked to provide feedback on the results of the Delphi method.

#### Consensus meeting

If consensus has not been reached after the 3-round Delphi or there is significant disagreement, a consensus meeting will be held with the objective of ratifying the outcomes for inclusion in the COS. The results from each round of the Delphi survey will be reviewed and participants will approve the outcomes that meet consensus criteria for inclusion and exclusion. Participants also will discuss the outcomes where there was lack of agreement. Outcomes where there was a lack of consensus during the Delphi process will be discussed and participants will anonymously vote for each outcome for inclusion and exclusion in the finalized COS. At the conclusion of the process, we should have identified what COS should be measured in clinical trials for BMS.

## Discussion

This protocol paper describes the multi-step process that will be used to develop a COS for BMS. At the time of writing, there is no published COS for BMS. A COS for the management of BMS will help improve the design, conduct, and reporting of future RCTs, with reduced outcome reporting bias and heterogeneity between studies. This will enhance the comparability of studies and allow pooling of data for meta-analyses. A finalized COS reporting guideline and explanatory document will be drafted as determined by the Delphi rounds and consensus meeting. These documents will be disseminated by high-impact publication and through relevant international meetings. The outcomes included will be relevant to a range of stakeholders including patients, healthcare professionals, researchers, and healthcare policy decision-makers.

## Supplementary information

**Additional file 1.** Core Outcome Set-STAndardised Protocol Items: the COSSTAP Statement.

## Data Availability

Not applicable
