# Correction to: IMMPACT-recommended outcome measures and tools of assessment in burning mouth syndrome RCTs: an international Delphi survey protocol

**DOI:** 10.1186/s13063-021-05183-y

**Published:** 2021-03-22

**Authors:** Barbara Carey, Arwa M. Farag, Cibele Nasri-Heir, Gary D. Klasser, Anura Ariyawardana, Milda Chmieliauskaite, Andrea Sardella, Charles R. Carlson, Craig S. Miller, Lina Mejia, Francis E. O’Neill, Ruy Albuquerque

**Affiliations:** 1 Oral Medicine Department, Guy’s and St. Thomas Hospital NHS Foundation Trust, King’s College London, London, UK; 2 Department of Oral Diagnostic Sciences, Faculty of Dentistry, King AbdulAziz University, Jeddah, Saudi Arabia; 3 Division of Oral Medicine, Department of Diagnostic Sciences, Tufts School of Dental Medicine, Boston, MA USA; 4 Department of Diagnostic Sciences, Center for Temporomandibular Disorders and Orofacial Pain, Rutgers School of Dental Medicine, The State University of New Jersey, Newark, NJ USA; 5 Department of Diagnostic Sciences, School of Dentistry, Louisiana State University Health Sciences Center, New Orleans, LA USA; 6 College of Medicine and Dentistry, James Cook University, Cairns, Queensland Australia; 7 Metro South Oral Health, Brisbane, Queensland Australia; 8 Department of Oral and Maxillofacial Medicine and Diagnostic Sciences, School of Dental Medicine, Case Western Reserve University, Cleveland, OH USA; 9 Department of Biomedical, Surgical and Dental Sciences, Unit of Oral Medicine, Oral Pathology and Gerodontology, University of Milan, Milan, Italy; 10 Orofacial Pain Clinic, College of Dentistry, University of Kentucky, Lexington, KY USA; 11 Department of Oral Health Practice, College of Dentistry, University of Kentucky, Lexington, KY USA; 12 College of Dental Medicine, Nova Southeastern University, Fort Lauderdale, FL USA; 13 Department of Oral Surgery, School of Dentistry, University of Liverpool, Liverpool, UK; 14 Department of Oral Medicine, Faculty of Dentistry, Oral Craniofacial Sciences, King’s College London, Floor 22, Guy’s Tower, London, SE1 9RT UK

**Correction to: Trials (2020) 21:711**

**https://doi.org/10.1186/s13063-020-04640-4**

Following publication of the original article [1], the authors expressed their preference for their first names to be displayed in full, not just by initials.
