# Supplementary material for: IMMPACT-recommended outcome measures and tools of assessment in burning mouth syndrome RCTs: an international Delphi survey protocol
Source: Trials. 2020 Aug 12;21:711. doi: 10.1186/s13063-020-04640-4 (PMC7425408; doi:10.1186/s13063-020-04640-4)
Supplement: Supplementary file 1 — Additional file 1. Core Outcome Set-STAndardised Protocol Items: the COSSTAP Statement. [file 13063_2020_4640_MOESM1_ESM.pdf]

Core Outcome Set-STAndardised Protocol Items: the COS-STAP Statement

TITLE/ABSTRACT			
Title	1a	Identify in the title that the paper describes the protocol for the planned development of a COS	Complete<br>P.1
Abstract	1b	Provide a structured abstract	Complete<br>P. 3-4
INTRODUCTION			
Background and objectives	2a	Describe the background and explain the rationale for developing the COS, and identify the reasons why a COS is needed and the potential barriers to its implementation	Complete<br>P. 5,6,7
	2b	Describe the specific objectives with reference to developing a COS	Complete<br>P. 9
Scope	3a	Describe the health condition(s) and population(s) that will be covered by the COS	Complete<br>P. 9
	3b	Describe the intervention(s) that will be covered by the COS	Complete<br>P. 9
	3c	Describe the context of use for which the COS is to be applied	Complete<br>P. 9,10
METHODS			
Stakeholders	4	Describe the stakeholder groups to be involved in the COS development process, the nature of and rationale for their involvement and also how the	Complete<br>P. 9, 11

Core Outcome Set-STAndardised Protocol Items: the COS-STAP Statement

		individuals will be identified; this should cover involvement both as members of the research team and as participants in the study	
Information sources	5a	Describe the information sources that will be used to identify the list of outcomes. Outline the methods or reference other protocols/papers	Complete<br>P11, 12
	5b	Describe how outcomes may be dropped/combined, with reasons	Complete<br>P 11,12,13,14
Consensus process	6	Describe the plans for how the consensus process will be undertaken	Complete<br>P 8
Consensus definition	7a	Describe the consensus definition	Complete<br>P12,13,16
	7b	Describe the procedure for determining how outcomes will be added/combined/dropped from consideration during the consensus process	Complete<br>P12,13,14
ANALYSIS			
Outcome scoring/feedback	8	Describe how outcomes will be scored and summarised, describe how participants will receive feedback during the consensus process	Complete<br>P 12,13,14
Missing data	9	Describe how missing data will be handled during the consensus process	Complete<br>P11
ETHICS and DISSEMINATION			

Core Outcome Set-STAndardised Protocol Items: the COS-STAP Statement

Ethics approval/informed consent	10	Describe any plans for obtaining research ethics committee/institutional review board approval in relation to the consensus process and describe how informed consent will be obtained (if relevant)	Complete P12
Dissemination	11	Describe any plans to communicate the results to study participants and COS users, inclusive of methods and timing of dissemination	Complete P15
ADMINISTRATIVE INFORMATION			
Funders	12	Describe sources of funding, role of funders	Complete P17
Conflicts of interest	13	Describe any potential conflicts of interest within the study team and how they will be managed	Complete P17
